# A Role for PCNA Ubiquitination in Immunoglobulin Hypermutation

**DOI:** 10.1371/journal.pbio.0040366

**Published:** 2006-10-24

**Authors:** Hiroshi Arakawa, George-Lucian Moldovan, Huseyin Saribasak, Nesibe Nur Saribasak, Stefan Jentsch, Jean-Marie Buerstedde

**Affiliations:** 1 Gesellschaft für Strahlen Forschung, Institute for Molecular Radiobiology, Neuherberg-Munich, Germany; 2 Max Planck Institute of Biochemistry, Department of Molecular Cell Biology, Martinsried-Munich, Germany; Scripps Research Institute, United States of America

## Abstract

Proliferating cell nuclear antigen (PCNA) is a DNA polymerase cofactor and regulator of replication-linked functions. Upon DNA damage, yeast and vertebrate PCNA is modified at the conserved lysine K164 by ubiquitin, which mediates error-prone replication across lesions via translesion polymerases. We investigated the role of PCNA ubiquitination in variants of the DT40 B cell line that are mutant in K164 of PCNA or in Rad18, which is involved in PCNA ubiquitination. Remarkably, the *PCNA^K164R^* mutation not only renders cells sensitive to DNA-damaging agents, but also strongly reduces activation induced deaminase-dependent single-nucleotide substitutions in the immunoglobulin light-chain locus. This is the first evidence, to our knowledge, that vertebrates exploit the PCNA-ubiquitin pathway for immunoglobulin hypermutation, most likely through the recruitment of error-prone DNA polymerases.

## Abstract 2

Vertebrate DT40 cells with a mutation in*PCNA* that prevents the protein's ubiquitylation and SUMOylation are sensitive to DNA damage and are dramatically deficient in AID-induced hypermutation.

## Introduction

Proliferating cell nuclear antigen (PCNA), a homotrimeric DNA-encircling protein, is the key target of the conserved ubiquitin-dependent *RAD6* pathway of post-replicative DNA repair [1]. If replication fork movement is stalled by a DNA lesion, cells can recruit translesion polymerases to bypass the lesion or initiate error-free repair by using the undamaged sister chromatid. Studies in the yeast Saccharomyces cerevisiae suggest that the switch from replicative to translesion DNA synthesis is mediated by PCNA ubiquitination catalyzed by the E2 ubiquitin-conjugating enzyme Rad6 and the E3 ubiquitin ligase Rad18 [1,2]. Whereas K164 of yeast PCNA can be modified either by mono- or poly-ubiquitin or by small ubiquitin-related modifier (SUMO) [1], only mono-ubiquitination of PCNA was observed after methyl methanesulfonate (MMS) treatment or ultraviolet (UV) irradiation of a human cell line [1,3,4]. Mono-ubiquitination of human PCNA requires the human Rad18 homologue and increases the affinity of PCNA for the translesion DNA polymerases Polη [3,4] and REV1 [5]. Studies in S. cerevisiae have shown that the lysine-to-arginine substitution at amino acid position 164 of PCNA *(PCNA^K164R^)* prevents ubiquitination, but does not interfere with the essential function of PCNA in replication [1]. K164 is also the target of RAD18-mediated PCNA ubiquitination in higher eukaryotes [1,3].

Immunoglobulin (Ig) hypermutation is a cell-type and locus-specific mutation activity, which diversifies the rearranged V(D)J segments of the Ig genes by random nucleotide substitutions. Ig hypermutation requires activation-induced deaminase (AID) [6], which most likely initiates hypermutation by cytosine deamination within the Ig loci [7,8]. The resulting uracils are recognized either by the uracil glycosylase UNG-2 or by mismatch repair factors leading to mutations at G/C and A/T bases, respectively [9]. *Polη*−deficient human and murine B cells [10–12], a *REV1-*disrupted mouse, and a DT40 mutant [13–15] show an altered spectrum and a decreased frequency of Ig mutations respectively, but it remains unclear, how translesion DNA polymerases are engaged for the Ig hypermutation pathway.

Because of its high ratio of targeted DNA integration [16], the chicken DT40 cell line has become a popular genetic system to study DNA repair [17] and AID-induced Ig gene diversification [18,19]. To clarify the role of PCNA ubiquitination in higher eukaryotes, we tested the effect of the *PCNA^K164R^* mutation alone, or in combination with *RAD18* or *REV1* gene disruptions in the chicken B cell line DT40. The analysis of the mutant cell clones indicate for the first time that Ig hypermutation specifically exploits the same mechanism that mediates DNA damage-induced mutagenesis.

## Results

### Generation of a Genomic *PCNA* K164 Mutant

**Figure 1:**
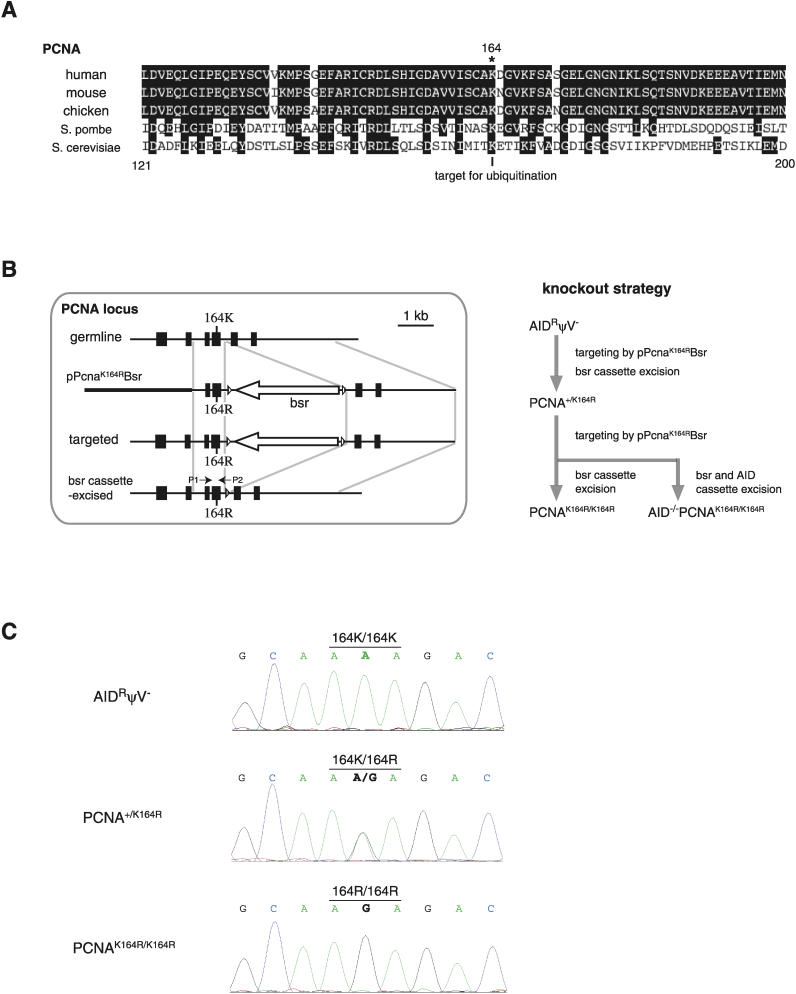
Site-Directed Mutagenesis of the *PCNA* Locus (A) Alignment of the human, mouse, chicken, *Schizosaccharomyces pombe,* and S. cerevisiae PCNA amino acid sequences. Amino acid 164 serving as the attachment site for ubiquitination in S. cerevisiae is marked by an asterisk. (B) A physical map of the *PCNA* locus and the *PCNA* mutagenesis construct, *pPcna^K164R^Bsr*. The targeting strategy of *PCNA* locus and the genealogy of the mutant clones are shown below and to the right, respectively. (C) Sequence chromatographs covering the *PCNA* codon 164 which was changed from AAA in the *AID^R^*ψ*V^−^* clone to AGA in the *PCNA^K164R/K164R^* clone.

The primary amino acid structure of PCNA indicates that the ubiquitin attachment site K164 is conserved from yeast to human (Figure 1A). Because cell-cycle regulated *PCNA* expression is likely to be important for normal cell proliferation, we preserved the physiologic expression control of the *PCNA* gene by introducing the K164R mutation into of endogenous locus. The DT40 variant *AID^R^*ψ*V* ^−^ [18] was chosen as the progenitor clone of the study, because it has the following properties: (i) it diversifies its rearranged Ig light-chain locus by hypermutation due to the deletion of the nearby pseudo *V* (ψ*V*) gene conversion donors; (ii) it expresses the *AID* gene as a floxed cDNA cassette; and (iii) it can be induced by tamoxifen to express Cre recombinase. After transfection of the *PCNA* mutagenesis construct *pPcna^K164R^Bsr* into *AID^R^*ψ*V* ^−^, a transfectant was identified that had integrated the construct targeted into one of the two *PCNA* alleles (Figure 1B). Excision of the floxed *Bsr* marker cassette (Figure 1B) produced the heterozygous mutant, *PCNA^+/K164R^*. To generate a homozygous *PCNA* mutant, *pPcna^K164R^Bsr* was retransfected into *PCNA^+/K164R^* and a transfectant having integrated the construct into the remaining wild-type allele was identified. Transient Cre induction in this transfectant yielded two clones—an *AID*-expressing, homozygous *PCNA* mutant, *PCNA^K164R/K164R^* —in which only the *Bsr* marker cassette had been excised and an AID negative control, *AID^−/−^PCNA^K164R/K164R^,* in which both the *Bsr* marker and the *AID* expression cassette had been removed. The status of the codon-164 mutations in the heterozygous and the homozygous mutant *PCNA* clones was confirmed by sequencing the exon 4 of the *PCNA* loci (Figure 1C). Both copies of either the *RAD18* or the *REV1* gene were disrupted by targeted integration in the *AID^R^*ψ*V^−^* and *PCNA^K164R/K164R^* clones yielding the single mutants *RAD18^−/−^* and *REV1^−/−^* as well as the double mutants *PCNA^K164R/K164R^ RAD18^−/−^* and *PCNA^K164R/K164R^ REV1^−/−^*. Notably, the *PCNA^K164R/K164R^* and *RAD18^−/−^* clones did not show a growth defect, compared to the *AID^R^*ψ*V^−^* progenitor clone in the absence of genotoxic stress. However, the *REV1* single mutant and the *REV1*/*PCNA^K164R/K164R^* double mutants had reduced cloning efficiencies and proliferated more slowly (unpublished data).

### Biochemical Analysis of PCNA Modifications

**Figure 2:**
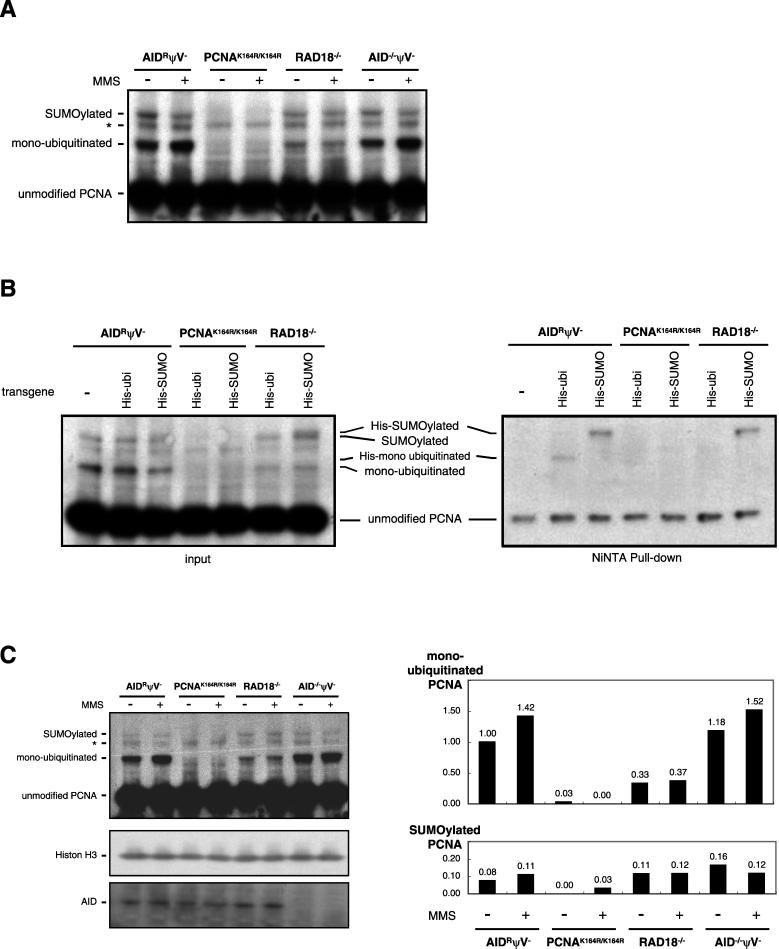
Ubiquitination and SUMOylation of PCNA (A) Cells were treated with or without MMS and were analyzed by immunoblotting using an monoclonal antibody to PCNA. The asterisk denotes a band reactive with PCNA antibodies, possibly corresponding to a PCNA modification independent of K164 and Rad18. (B) Analysis of clones stably transfected with His-tagged ubiquitin or SUMO-1 expression vectors. Whole cell lysates (left) and lysates after NiNTA chromatography (right) are shown. The positions expected for unmodified, mono-ubiquitinated, and SUMOylated PCNA are indicated by lines. Due to the low residual level of PCNA ubiquitination in the *RAD18* mutant, this modification could not be detected by pull-downs. The bands at the bottom represent low levels of unmodified PCNA unspecifically bound to the beads. (C) Quantification of mono-ubiquitinated and SUMOylated PCNA, histone H3, and AID by immunoblotting. Cells were treated with or without MMS, and immunoblotted using monoclonal antibodies to PCNA (left upper), histone H3 (left middle), and AID (left lower). The values for mono-ubiquitinated and SUMOylated PCNA given in the right hand graphs were calculated as described in the Materials and Methods.

Next, we probed cell lysates from untreated and MMS-treated cells for PCNA modifications by immunoblotting (Figure 2A). In addition to unmodified PCNA, *AID^R^*ψ*V^−^* cells showed protein species that had mobilities corresponding to mono-ubiquitinated and SUMOylated PCNA (Figure 2A). Whereas mono-ubiquitination of PCNA was detectable in yeast and HeLa cells only in the presence of DNA damaging agents [1], mono-ubiquitinated PCNA was observed in DT40 cells even in the absence of MMS. Mono-ubiquitination or SUMOylation of PCNA was not affected by the absence of AID expression in *AID^−/−^*ψ*V^−^*cells (Figure 2A). This is not surprising, because PCNA ubiquitination is likely to play an important role for general DNA repair of the genome, and any increase related to the processing of AID-induced DNA lesions in the Ig loci is unlikely to be detectable against this background.

To verify the identity of these protein species, cells were transfected by cDNA expression constructs encoding His-tagged versions of either ubiquitin or SUMO. A comparison of whole-cell lysates and cell lysates purified by NiNTA pulldown confirmed the identity of the bands assigned to mono-ubiquitinated and SUMOylated PCNA (Figure 2B). To quantify the amounts of mono-ubiquitinated and SUMOylated PCNA more precisely in the different cell samples, Western blots were repeated using in parallel an antibody to PCNA and an antibody to histone (Figure 2C, left). The signals obtained by the later antibody serve a loading control. This analysis showed, as expected, that mono-ubiquitination of PCNA was induced by MMS, and both mono-ubiquitinated and SUMOylated PCNA species were absent in *PCNA^K164R/K164R^* cells (Figure 2A and 2C, right). Whereas the signal for SUMOylated PCNA was not affected in *RAD18^−/−^* cells, the signal for mono-ubiquitinated PCNA was significantly reduced and apparently not inducible by MMS. An *AID* negative control clone, *AID^−/−^*ψ*V^−^*, showed a pattern identical to the progenitor *AID^R^*ψ*V^−^*. The levels of AID expression as determined by an antibody to AID did not vary among the different cell clones (Figure 2C). Taken together, the results indicate that the *PCNA^K164R^* mutation prevents PCNA ubiquitination and SUMOylation, whereas the *RAD18* gene disruption decreases, but does not abolish mono-ubiquitination of PCNA in DT40.

### The *PCNA^K164R^* Mutant Is Sensitive to DNA Damage

**Figure 3:**
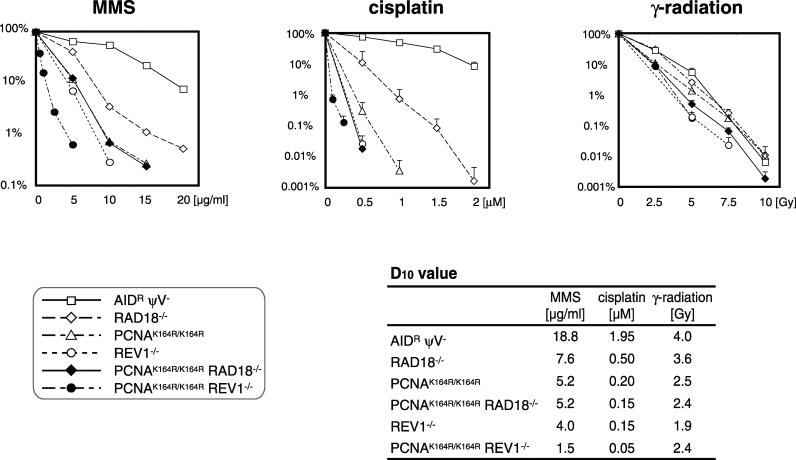
Colony Survival Curves after Exposure to DNA-Damaging Agents The values of DNA damaging agents, which give 10% cell viability, are also summarized (*D*_10_ values).

Because PCNA ubiquitination is known to be crucial for bypass replication across DNA lesions in S. cerevisiae [1], we asked whether the same mechanism operates in vertebrates as well. To investigate the role of PCNA modification for DNA damage tolerance, the survival of the different clones was determined after exposure to the DNA alkylating agent MMS, the DNA interstrand cross-linking agent cisplatin, and γ radiation (Figure 3). Compared to the progenitor cells, *PCNA^K164R/K164R^* cells are highly sensitive to MMS and cisplatin, but only mildly sensitive to γ radiation. The survival of *RAD18^−/−^* cells after exposure to all three types of genotoxic stress is lower than of *AID^R^*ψ*V^−^* cells, but higher than of *PCNA^K164R/K164R^* cells (Figure 3). The *PCNA^K164R/K164R^* *RAD18^−/−^* double mutant shows similar, or slightly lower, survival rates than the *PCNA^K164R/K164R^* single mutant. However, *PCNA^K164R/K164R^* *REV1^−/−^* cells proliferate poorly even in the absence of genotoxic stress and showed significantly lower survival rates than *REV1^−/−^* cells. Together, these findings strongly suggest that PCNA ubiquitination at the conserved K164 is crucial for DNA damage tolerance also in vertebrates, demonstrating that the RAD6 pathway is conserved across species and that PCNA is the conserved target.

### The *PCNA^K164R^* Mutant Is Defective in Hypermutation at the Ig Locus

**Figure 4:**
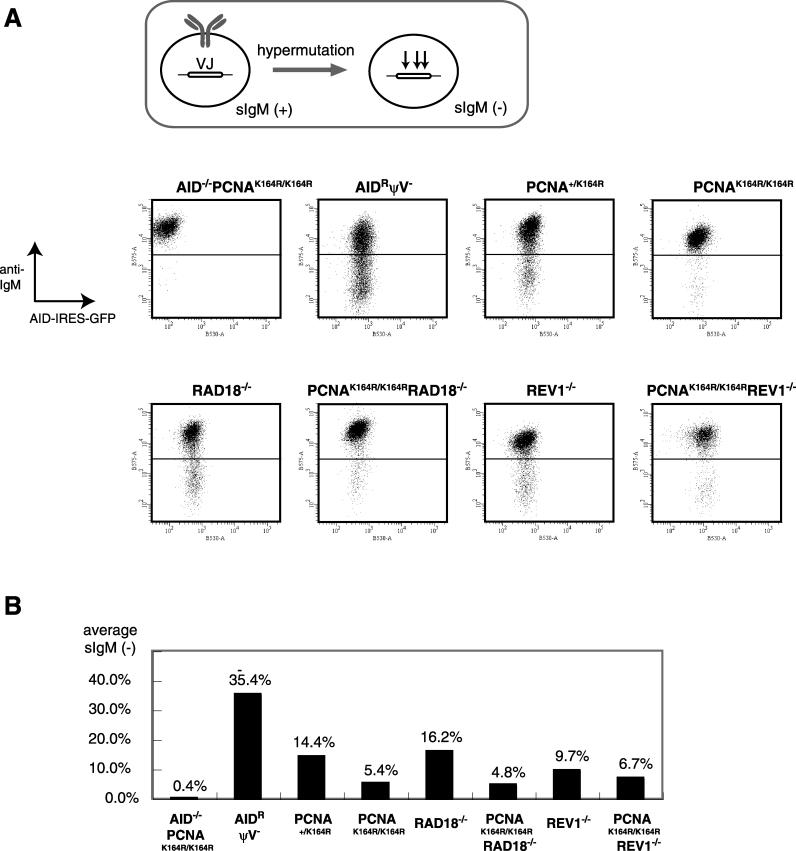
FACS Analysis of Ig Hypermutation Activity (A) FACS profiles of representative subclones derived from a sIgM (+) cell after staining with a monoclonal antibody to IgM. (B) The average percentages of events falling into sIgM (−) gates based on the measurement of 24 subclones are shown by graph.

All clones included in the study were cell-surface Ig positive [sIg(+)], allowing the detection of deleterious Ig light-chain mutations by loss of sIg expression [18]. To compare the mutation rates, fluorescence activated cell sorting (FACS) was performed for 24 subclones of each of the control and mutant clones 2 wk after subcloning (Figure 4). Whereas subclones of the nonhypermutating control *AID^−/−^ PCNA^K164R/K164R^* show on average 0.4% of the total events in the sIg(*−*) gate, subclones of the *AID^R^*ψ*V^−^* progenitor show 35.4%. In contrast, the average percentages of sIg(*−*) events for subclones of the heterozygous *PCNA^+/K164R^* and the homozygous *PCNA^K164R/K164R^* are only 14.4% and 5.4%, respectively. The averages for subclones of *RAD18^−/−^* and *REV1^−/−^* are 16.2% and 9.7%, respectively and the *PCNA^K164R/K164R^ RAD18^−/−^* and *PCNA^K164R/K164R^ REV1^−/−^* double mutants behave similar to the *PCNA^K164R/K164R^* single mutant. This analysis indicates that the *PCNA^K164R^* mutation decreases the frequency of deleterious Ig mutations about 7-fold, whereas the reduction is only about 2-fold in the *RAD18* knockout and about 3- to 4-fold in the *REV1* knockout. A reduction of sIg(*−*) cells is already detected for the heterozygous *PCNA^+/K164R^* subclones suggesting a dose-dependent effect. All mutant clones are derived from the same *AID^R^*ψ*V^−^* progenitor, and the expression levels of *AID* are not expected to vary among AID-positive clones. Because green fluorescent protein (GFP) is expressed together with the *AID* transgene (*AID*-*IRES*-*GFP*), constant levels of AID in all those clones were further confirmed by measuring GFP expression on the *x*-axis of the FACS plots.

**Figure 5:**
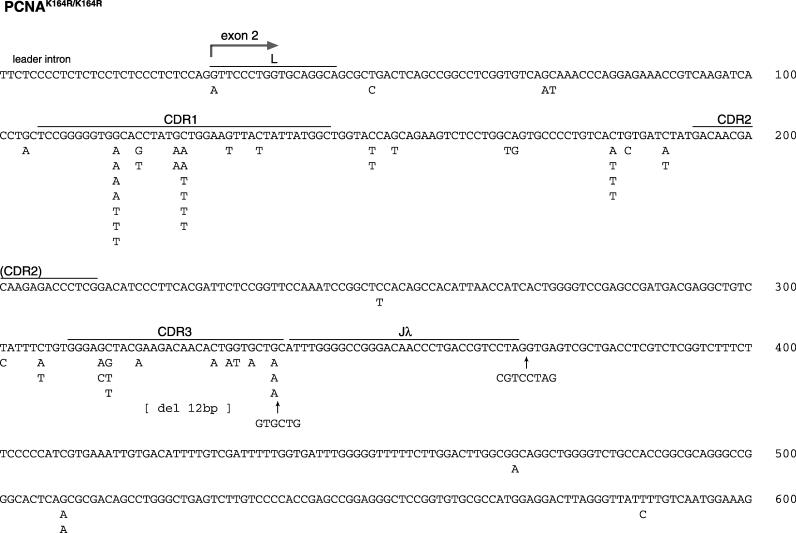
Ig Hypermutation of the *PCNA^K164R/K164R^* Clone Ig light-chain sequence variation in the *PCNA^K164R/K164R^* clone. All sequence differences in the region from the first intron to the J-C intron are shown relative to the rearranged light-chain consensus sequence of the *AID^R^*ψ*V^−^* precursor clone. The position of complementary determining regions CDR1, CDR2, and CDR3 and that of Jλ are indicated.

**Figure 6:**
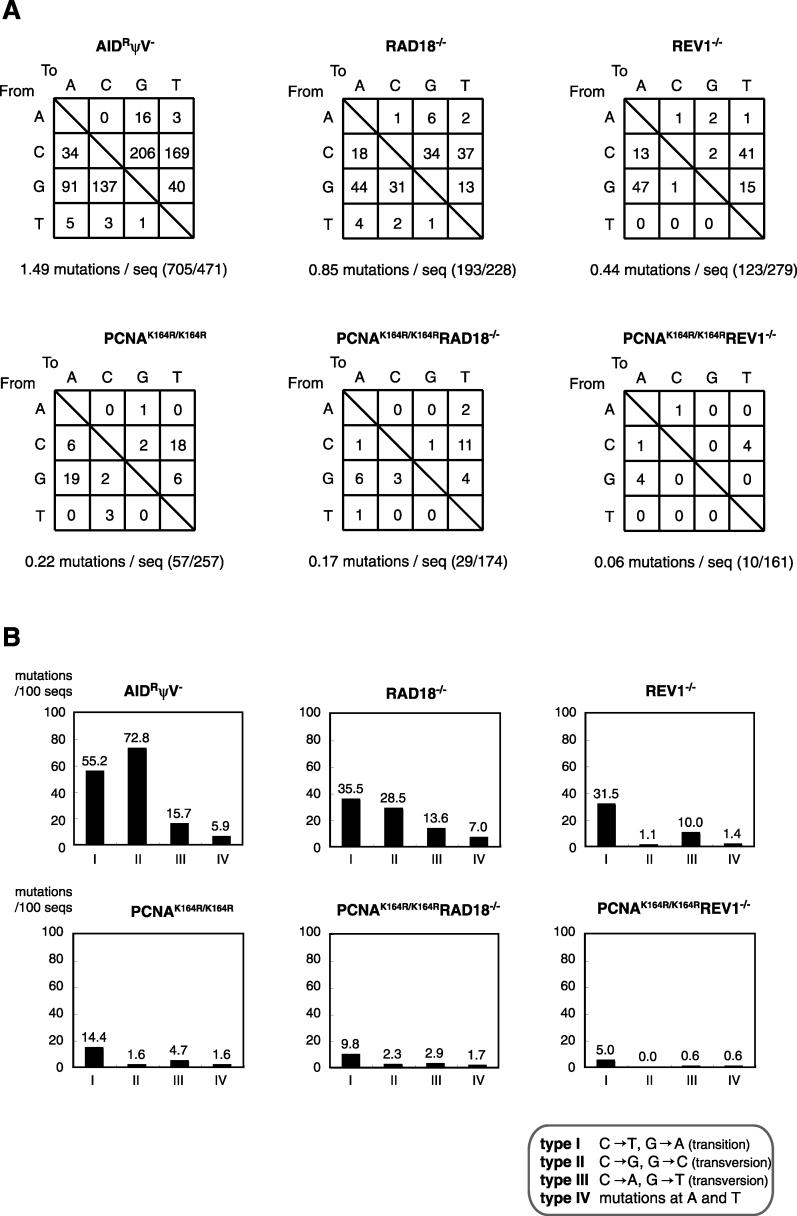
Mutation Spectrum (A) Frequencies of particular nucleotide substitutions within light-chain gene. (B) A graphical view showing the frequencies of different types of mutations per hundred sequences.

To directly analyze the mutation frequencies and spectra, light-chain VJ segments were sequenced from several subclones of each control and mutant clone 6 wk after subcloning. When aligned to a consensus sequence of the rearranged Ig light-chain gene, mutations from *PCNA^K164R/K164R^* (Figure 5) and from *REV1^−/−^* (unpublished data) cells are similarly distributed as mutations from *AID^R^*ψ*V^−^* cells [18]. However, as expected from the analysis of sIg loss rates, *RAD18^−/−^, REV1^−/−^,* and *PCNA^K164R/K164R^* cells yield about 2-, 3-, and 7-fold fewer of mutations per sequence, respectively, than *AID^R^*ψ*V^−^* cells do (Figure 6A). Whereas *PCNA^K164R/K164R^ RAD18^−/−^* cells have a similar frequency of mutations as *PCNA^K164R/K164R^* cells, the *PCNA^K164R/K164R^ REV1^−/−^* double mutant shows even fewer mutations than the *PCNA^K164R/K164R^* single mutant.

All types of mutations are reduced in *PCNA^K164R/K164R^* cells compared to *AID^R^*ψ*V^−^*cells, but the most pronounced decrease is seen for C-to-G and G-to-C transversions (type II mutations in Figure 6B). Interestingly, the same type of mutations are moderately reduced in *RAD18^−/−^* cells and strongly reduced in *REV1^−/−^* cells. *PCNA^K164R/K164R^* and *PCNA^K164R/K164R^ RAD18^−/−^* cells show similar mutation spectra and mutation frequency. The mutation spectrum of *PCNA^K164R/K164R ^ REV1^−/−^* cells appears to be similar to that of *PCNA^K164R/K164R^* cells. Only three mutations—possibly PCR errors—were found in 89 light-chain VJ segments from *AID^−/−^ PCNA^K164R/K164R^* cells, indicating that AID is required for the low Ig mutation activity still present in the *PCNA* mutant (unpublished data).

## Discussion

This study demonstrates that the *PCNA^K164R^* single-codon substitution causes marked sensitivity to genotoxic stress and a strong decrease in Ig hypermutation in the DT40 cell line. Although the mutation prevents mono-ubiquitination as well as SUMOylation, the observed phenotype is most likely due to the lack of ubiquitination. This is consistent with the finding that the *RAD18* knockout, which does not affect PCNA SUMOylation but decreases PCNA ubiquitination, shows a similar, though more modest, phenotype compared to the *PCNA^K164R^* mutation. The results indicate that PCNA ubiquitination has not only preserved its role for DNA repair from yeast to higher eukaryotes, but has been exploited additionally for Ig diversification in vertebrate B cells. It is currently difficult to assert the role of the PCNA SUMOylation, because the Srs2 protein, which is recruited by SUMOylated PCNA in S. cerevisiae [20,21] is not conserved during evolution. Similar to the situation in yeast, the DT40 *PCNA^K164R^* mutant does not exhibit any obvious defects in proliferation.

Studies in yeast [1] and evidence from human and mouse cells [3,4] suggest that PCNA mono-ubiquitination induces a switch from replicative to translesion DNA synthesis [3]. The most straightforward explanation of the *PCNA^K164R^* phenotype in DT40 is likewise a defect in the recruitment of error-prone translesion DNA polymerases. One of the polymerases activated by PCNA ubiquitination seems to be Rev1, because *PCNA^K164R/K164R^* and *REV1^−/−^* cells show similar sensitivities to DNA-damaging reagents and a similar decrease in C-to-G and G-to-C mutations. The selective decrease of this type of hypermutation may reflect the deoxycytidyl transferase activity of Rev1, which would add cytosine opposite to an abasic site in the template strand [22]. These ideas are supported by the recent observations that mono-ubiquitinated PCNA can recruit REV1 [5] and that a *REV1* mutant in which the deoxycytidyl transferase activity is selectively inactivated does not rescue the Ig hypermutation defect seen in DT40 after *REV1* disruption [23]. Other types of Ig hypermutations are significantly decreased in the *PCNA^K164R/K164R^* cells, but not in *REV1^−/−^* cells, suggesting that PCNA ubiquitination directly activates other translesion DNA polymerases apart from Rev1. Vice versa, the low viability and increased DNA damage sensitivity of *PCNA^K164R/K164R^ REV1^−/−^* cells indicates that Rev1 fulfills some functions independent of PCNA ubiquitination. C-to-T and G-to-A transitions predominate among the mutations still detected in *PCNA^K164R/K164R^* cells. Although these mutations could be due to an error-prone pathway operating independently of PCNA ubiquitination, they may also reflect AID-induced uracils, which have escaped excision by UNG-2 and have paired with adenines during replication.

The remaining low level of PCNA mono-ubiquitination in DT40 *RAD18^−/−^* cells, recently also reported by another group [24], is surprising given the fact that Rad18 is entirely responsible for PCNA mono-ubiquitination in S. cerevisiae. The *RAD18* knockout construct used in the studies most likely generates a *RAD18* null mutation [25], although it does not delete the *RAD18* RING finger coding sequence. To rule out that the analyzed *RAD18* mutant still possessed enzymatic activity, we generated a second *RAD18* mutant in which the RING finger-coding region is deleted. Analysis of this new mutant confirmed the persistence of low-level PCNA ubiquitination seen in the first *RAD18* mutant (unpublished data). These data point to the presence of a Rad18-independent back-up pathway of PCNA ubiquitination in vertebrate cells. A possible candidate for an E3 ligase involved in PCNA ubiquitination in *RAD18^−/−^* cells may be the gene product of *FANCL* [26]. The residual PCNA ubiquitination may explain the milder DNA damage sensitivity and the higher Ig mutation rate of *RAD18^−/−^* cells compared to *PCNA^K164R/K164R^* cells. Whereas it was initially reported that *RAD18* disruption in wild-type DT40 does not affect Ig hypermutation [27], we detected a ~2-fold reduction of Ig hypermutation in pseudogene deleted *RAD18^−/−^* cells, and another group recently reported a strong decrease in hypermutation activity [28]. We believe this discrepancy may be caused by the difficulty to accurately measure Ig hypermutation in wild-type DT40, which diversifies its Ig genes predominantly by gene conversion.

If ubiquitinated PCNA functions as a link to the recruitment of error-prone polymerases during Ig hypermutation, it remains an intriguing question how it is coupled to upstream events in the hypermutation process. The DNA editing model assumes that AID first deaminates cytosine to uracil and that the resulting uracil is then excised by UNG-2 [7]. A comparison of the mutation frequencies in *UNG-*disrupted and ψ*V-*deleted DT40 suggests that about one in seven AID-induced uracils is converted into a mutation [8]. This high mutation rate suggests that the abasic sites produced by uracil excision are not repaired by the standard base excision repair, but are deliberately channeled into error-prone translesion synthesis. One of the possibilities is that UNG-2 is recruited by PCNA [29] and excises AID-induced uracils shortly before DNA synthesis, thereby precluding the possibility of base excision repair. Another possibility is that the DNA lesions produced by the combined action of AID and UNG are for some reason, perhaps by protein attachment or by another type of modification, guarded from faithful repair until they encounter the PCNA clamp.

## Materials and Methods

### Target disruption of the *RAD18* and *REV1* genes.

The *RAD18* knockout constructs, which delete codons 163–182 of the *RAD18* gene, were obtained from Dr. Shunichi Takeda (Graduate School of Medicine, Kyoto University, Kyoto, Japan). The *REV1* knockout constructs were designed to delete *REV1* codons 119–407 by targeted integration. These constructs were transfected into the *AID^R^*ψ*V^−^* and *PCNA^K164R/K164R^* clones in a stepwise manner to generate the following homozygous knockout clones: *RAD18^−/−^, PCNA^K164R/K164R^ RAD18^−/−^, REV1^−/−^,* and *PCNA^K164R/K164R^ REV1^−/−^* (Figure S1). New *RAD18* knockout constructs were designed to delete *RAD18* codons 1–90, which include the whole RING finger motif. These constructs were transfected into the *AID^R^* clone, yielding the second *RAD18^−/−^* mutant.

### Site-directed mutagenesis of *PCNA* locus.

The cDNA sequence AB053163 in the public databases includes the full-length open reading frame of the chicken *PCNA* sharing 94% identity and 97% homology with the codons of human *PCNA*. Comparison of the cDNA sequence to the chicken genome sequence [30] revealed the exon-intron structure of the *PCNA* locus on chromosome 22. The sequence intended as the 5' arm of the *PCNA* targeting construct was first amplified by PCR from DT40 genomic DNA as two fragments using overlapping primers that included a point mutation to change codon 164 from lysine to arginine. The two fragments were then combined by chimeric PCR to yield the 5' targeting arm including the codon 164 mutation. The 3' targeting arm was amplified by PCR using genomic DNA from DT40 as template. Both arms were cloned upstream and downstream of the floxed *Bsr* resistance marker [31], yielding the PCNA mutagenesis construct *pPcna^K164R^Bsr*. The construct was linearized by *Not*I before transfection. Cell culture, transfection, selection of stable transfectants, and marker recycle by transient Cre induction were performed as previously described [32]. Transfectants having integrated the construct by targeted integration were identified by PCR using a primer derived from the PCNA locus upstream of the 5' targeting arm together with a primer derived from the *Bsr* gene. To confirm the status of the point mutation at codon 164, the region surrounding exon 4 was amplified by PCR from genomic DNA of the *AID^R^*ψ*V^−^, PCNA^+/K164R^* and *PCNA^K164R/K164R^* clones and directly sequenced without cloning. Apart from the P2 and P3 primers, which were used for sequencing, all other primers were used for the verification of gene targeting by PCR.

The primer sequences are as follows: B1, CGATTGAAGAACTCATTCCACTCAAATATACCC; G1, TGTTGATATCCCGCAAGATACCTGGATTGA; M1, AGCTTGGATAACTTCGTATAGCATACATTATACGAACGGTAGGG; P1, GGGGGATCCTAGTTGTTTGAATGACTGCATGCAC; P2, GATCAGAGAAATACCAGATCCAGTGACC; P3; TGCACTGTTCTTAGGACAGCATGTTTTCCT; P4, TAGTGTTTGAAGCACCAAGTGAGTGG; P5 CAAACCCCACAGCTAAGAAAGTGCC; R1, GTTTGCATATATGTGTGTGTGTGCATATGT; R2, TCATAGCAGCAGCAGCAGTTCTCCAGAGGC; R3, AGATGGATGCCACAGACTGACGCACACAGC; V1, ATGATGTTGCATGGAGGTCAATACCATGT; V2, GCGTCTCCTCTCGCACATTCCGTATCAGCT; and V3, GAGTTAGGTAATTGTAGCTCCATCCTCAAC.

### PCNA modifications.

DT40 cells were incubated in 0.02% MMS for 2 h. Total cell lysates were sonicated, separated on 4%–12% Bis-Tris gels, and immunoblotted with PC10 monoclonal antibodies to PCNA (Abcam, Milton Road, Cambridge, United Kingdom), 3H1 histone H3 (Cell Signaling Technology), and L7E7 AID (Cell Signaling Technology, Beverly, Massachusetts, United States). For NiNTA purification, the coding sequences for His-tagged human ubiquitin [1] or human SUMO1 [33] were cloned under chicken β-actin promoter, and stably transfected into the *AID^R^*ψ*V^−^, RAD18^−/−^,* and *PCNA^K164R/K164R^* clones. NiNTA chromatography was performed as described [1], and samples were immunoblotted with the PC10 antibody. The signals for mono-ubiquitinated and SUMOylated PCNA were quantified from the gel image of the Western blot shown in Figure 2C. First, the lowest respective value for mono-ubiquitinated and SUMOylated PCNA was assumed to be the background noise, and this value was subtracted. In the second step, the signals for each sample on the anti-PCNA Western blot at the positions of mono-ubiquitinated and SUMOylated PCNA, respectively, were normalized according to the signals obtained from the anti-histone Western blot to account for sample loading variation. In the last step all values were normalized to the value of mono-ubiquitinated PCNA in non-MMS treated *AID^R^*ψ*V^−^* cells, which was taken as 1.00.

### Colony survival assays.

Colony survival on methylcellulose-containing medium was performed as described [34]. Cisplatin and methylmethane sulphonate were obtained from Sigma (St. Louis, Missouri, United States). Cs^137^ was used for γ radiation resource. Each curve is derived from two to three separate experiments.

### Ig reversion assay.

Subcloning, antibody staining, flow cytometry, and quantification of sIgM expression has been described previously [32].

### Ig gene sequencing.

To minimize PCR-introduced artificial mutations, *Pfu*Ultra hotstart polymerase (Stratagene, La Jolla, California, United States) was used for amplification prior to sequencing. Long-range PCR and sequencing were performed as previously described [32]. To minimize the fluctuation effects, light-chain gene sequences were determined from several subclones of each mutant and control clone. The reference sequence for the mutation analysis was deduced by comparing the different sequences from each subclone. Sequences from *AID^R^*ψ*V^−^* subclones were pooled with sequences previously obtained under the same conditions [8,18] to establish a larger dataset to which the results from the *PCNA, REV1,* and *RAD18* mutants could be compared.

## Supporting Information

Figure S1 Gene Targeting Strategies and Screenings for Clones Having Undergone Targeted Integration Events Physical maps of the loci, the targeting vectors, and the targeted alleles are shown for *PCNA* (A), *RAD18* (B), and *REV1* (C), respectively. The position and orientation of the primers used for the screening by long-range PCR are indicated. The identification of the desired clones relied on the appearance of new PCR fragments as well as the disappearance of germline fragments. λ *Hin*dIII/ϕX *Hae*III was used as size marker.
(366 KB PDF)

### Accession Numbers

The Swiss-Prot (http://www.ebi.ac.uk/swissprot) accession numbers for the sequences displayed in Figure 1 are human (P12004), mouse (P17918), chicken (AB053163), Schizosaccharomyces pombe (Q03392), and S. cerevisiae (NP_009645).

## Abbreviations

AID: activation induced deaminase
Ig: immunoglobulin
MMS: methyl methanesulfonate
PCNA: proliferating cell nuclear antigen
sIg: cell-surface Ig
SUMO: small ubiquitin-related modifier
UNG: uracil DNA glycosylase
ψ*V*: pseudo variable
