# Supplementary material for: A Role for PCNA Ubiquitination in Immunoglobulin Hypermutation
Source: PLoS Biol. 2006 Oct 24;4(11):e366. doi: 10.1371/journal.pbio.0040366 (PMC1618868; doi:10.1371/journal.pbio.0040366)
Supplement: Supplementary file 1 [file pbio.0040366.sg001.pdf]

A

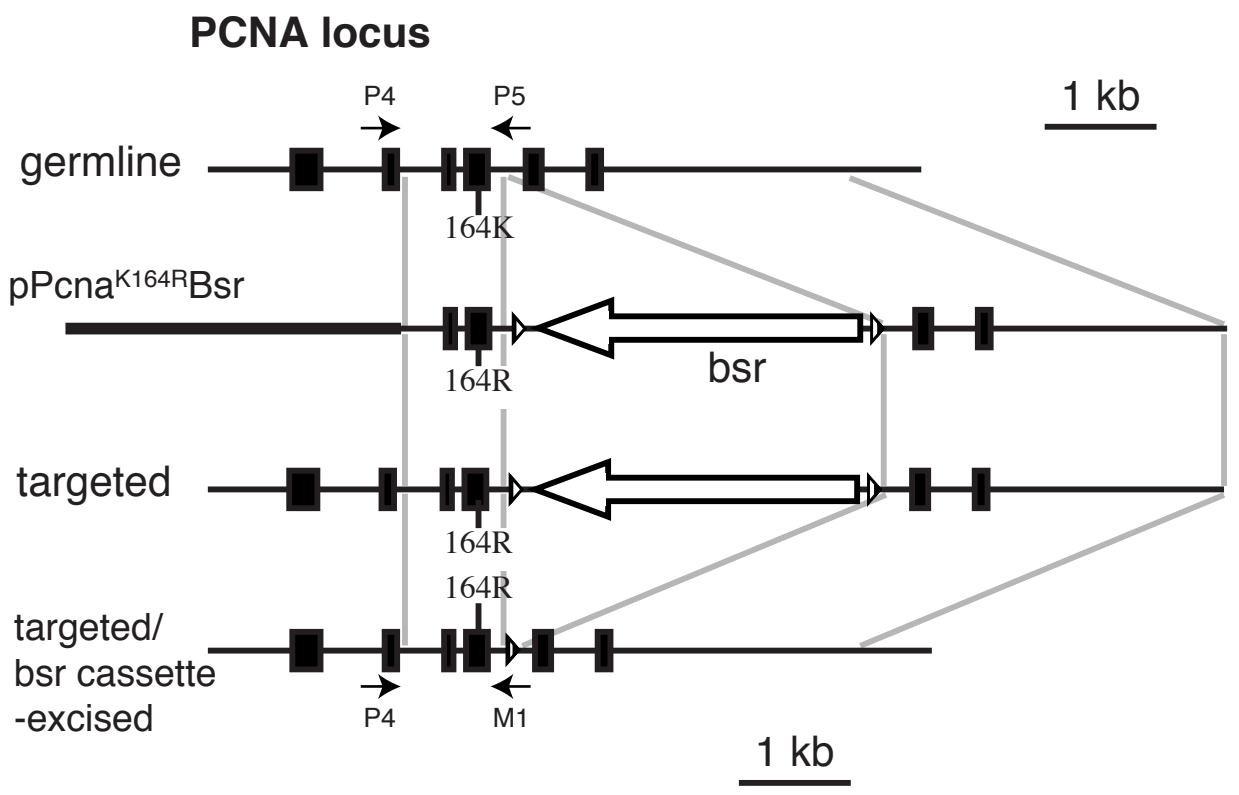

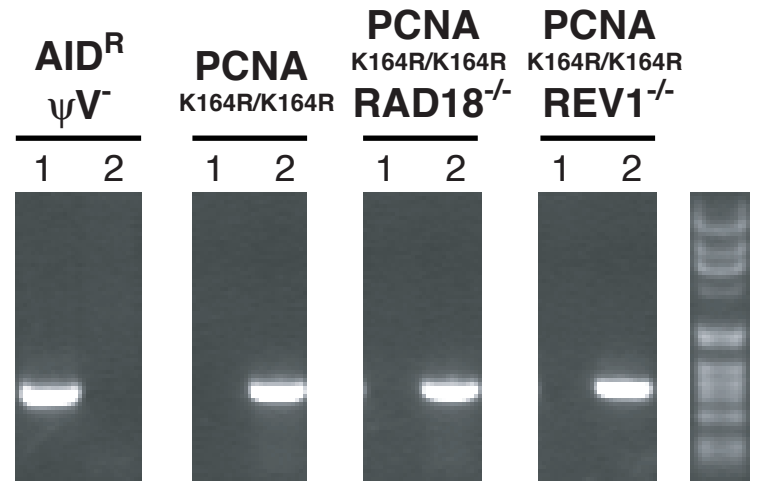

		primer pair	PCR product
1	germline control	P4/P5	1.0 kb
2	target screening	P4/M1	1.0 kb

B

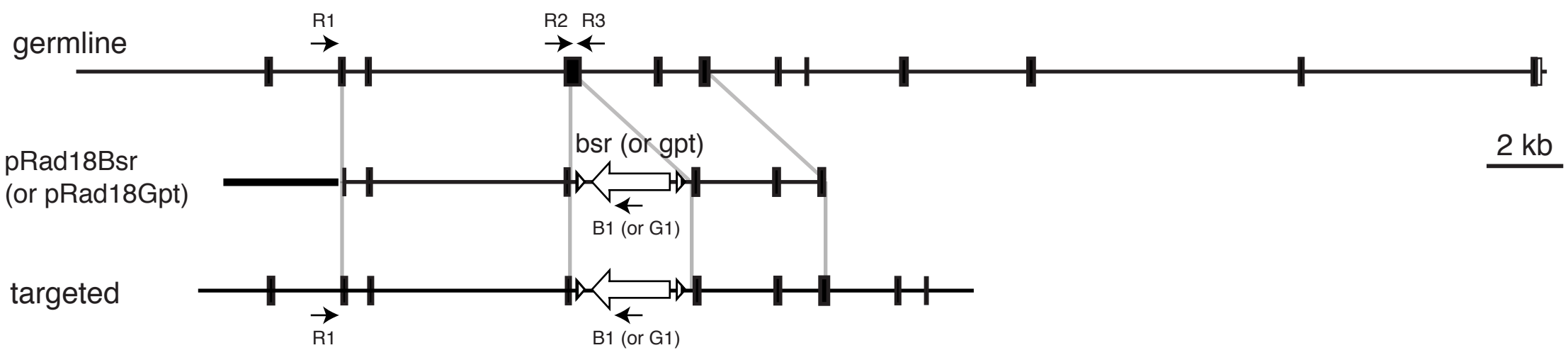

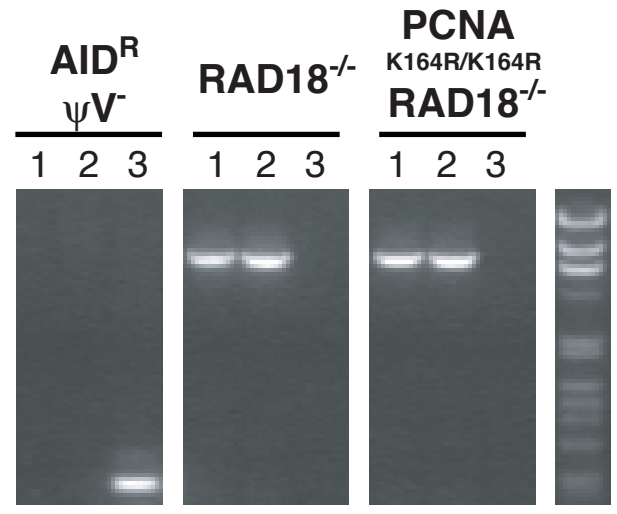

		primer pair	PCR product
1	bsr-target screening	R1/B1	7.3 kb
2	gpt-target screening	R1/G1	7.0 kb
3	germline control	R2/R3	0.2 kb

C

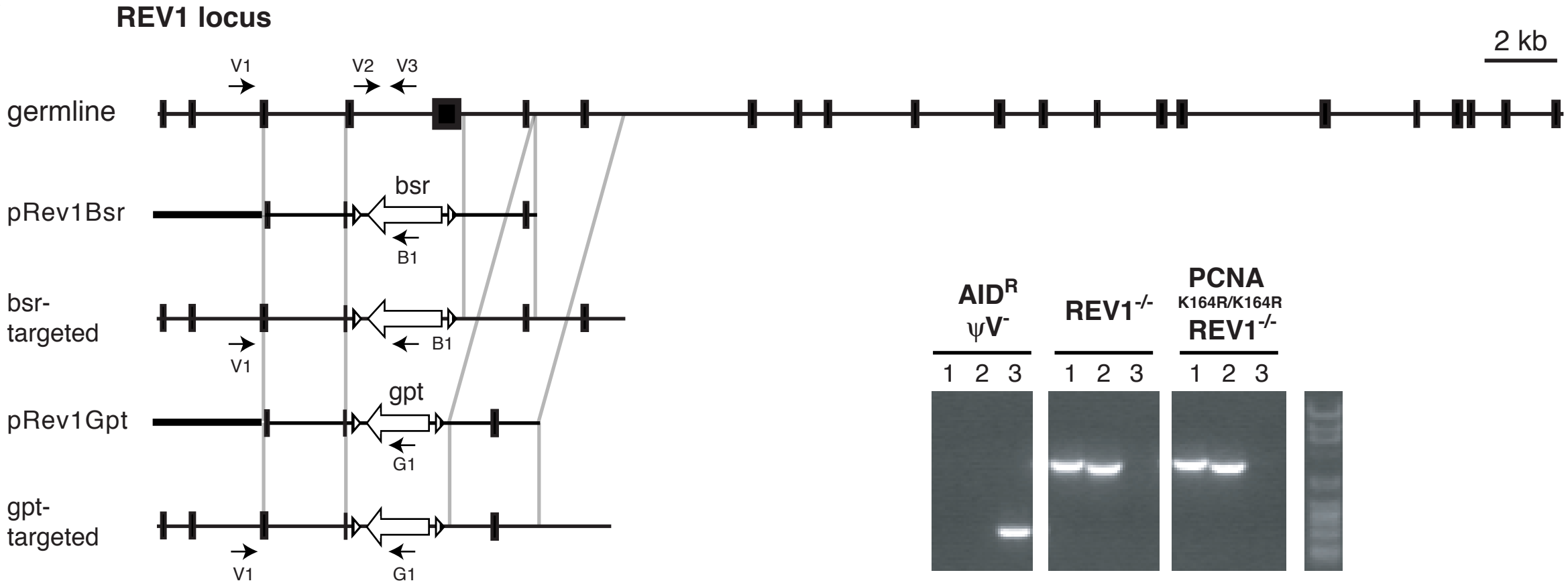

		primer pair	PCR product
1	bsr-target screening	V1/B1	3.4 kb
2	gpt-target screening	V1/G1	3.1 kb
3	germline control	V2/V3	0.7 kb
